# Early intervention in Hirschsprung’s disease: effects on enterocolitis and surgical outcomes

**DOI:** 10.1186/s12887-024-04956-z

**Published:** 2024-07-26

**Authors:** Yunhan Zhang, Xiao Xiang, Xunfeng Li, Wei Feng, Zhenhua Guo

**Affiliations:** 1 Department of neonatal surgery, National Clinical Research Center for Child Health and Disorders, Ministry of Education Key Laboratory of Child Development and Disorders, Chongqing Key Laboratory of Structural Birth Defect and Reconstruction, Children’s Hospital of Chongqing Medical University, Chongqing, China; 2 20, Jinyu Road, Yubei District, Chongqing, 400025 China

**Keywords:** Hirschsprung’s disease, Age, Enterocolitis, Functional outcomes

## Abstract

**Background:**

The timing of surgical intervention for Hirschsprung’s disease (HSCR) has been a topic of continued discussion. The objective of this study was to evaluate the significance of age at surgery in the management of HSCR by conducting a comparative analysis of the correlation between surgical age and midterm outcomes.

**Methods:**

We conducted a retrospective analysis of children with HSCR who underwent one-stage laparoscopic assisted pull-through surgery with modified Swenson technology at our hospital between 2015 and 2019. The study population was stratified into two groups based on surgical age: patients who underwent surgery within a period of less than 3 months and those who underwent surgery between 3 and 12 months. The basic conditions, complications at 3–7 years after surgery, anal function (Rintala scale) and quality of life (PedsQLTM4.0) were compared between the groups.

**Results:**

A total of 235 children (196 males and 39 females) were included in the study. No statistically significant differences in postoperative bowel function (*P* = 0.968) or quality of life (*P* = 0.32) were found between the two groups. However, there was a significant reduction in the incidence of Hirschsprung-associated enterocolitis (HAEC) among individuals under the age of three months prior to undergoing surgical intervention (69.1%) compared to the incidence observed postsurgery (30.9%). This difference was statistically significant (*P* < 0.001).

**Conclusion:**

In the current study, the age at which surgery was performed did not exhibit a discernible inclination towards influencing mid-term anal function or quality of life. Early surgical intervention can effectively diminish the occurrence of HAEC, minimize the extent of bowel resection, and expedite the duration of the surgical procedure.

## Introduction

Hirschsprung’s disease (HSCR) is a multifaceted genetic disorder characterized by a deficiency of intrinsic ganglion cells in the submucosal and myenteric plexuses of the gastrointestinal tract, which frequently results in neonatal intestinal obstruction [1]. Once HSCR is diagnosed, most children require surgical treatment. The primary approach involves the surgical removal of the section of the intestine where enteric neurons are absent, followed by reconstruction of the digestive tract [2].

Since the successful performance of the first primary pull-through without a protective colostomy in neonates was reported by So et al. [3], pediatric surgeons have been considering the appropriate timing of surgery when evaluating the conditions of patients. M. Carcassonne [4] and his team followed 32 children who underwent one-stage definitive surgery at less than 3 months of age for 2–10 years with three different procedures (Soave, Swenson, and Duhamel), and the results showed that all of them not only were considered clinically cured but also had good anal function. This is the first study to support the safety and effectiveness of one-stage transanal endorectal pull-through (TEPT) in children under 3 months of age. In the early 20th century, Weidner and Waldhausen [5] described the experience of 15 children with HSCR who underwent the Swenson procedure at a median age of approximately 1 month, which illustrated the safety of one-stage surgery for infants. However, the results have differed among various studies on the postoperative follow-up of children with HSCR. Additionally, some of them have shown poorer functional outcomes in infants at younger ages [6, 7]. Others have shown that a younger age of surgery corresponds to a better functional evaluation, especially for infants undergoing TEPT [8]. However, some scholars believe that postoperative functional prognosis is irrelevant to age at surgery [9–11].

Hence, the optimal timing for surgical intervention in patients with HSCR has been a topic of continuous scholarly discourse, particularly when considering recent advancements in diagnostic and minimally invasive surgical approaches, which have led to a trend towards performing surgery at a younger age. To date, there is a dearth of research examining the mid-term effects of early surgical intervention on defecation function and quality of life following laparoscopic-assisted transanal endorectal pull-through in infancy. This study aimed to explore the benefits and drawbacks of early intervention for treating HSCR in infants. This was accomplished by conducting a retrospective analysis comparing the anal function, quality of life, and postoperative complications of children who underwent one-stage laparo-scopic assisted pull-through surgery with modified Swenson technology before and after reaching 3 months of age. The ultimate goal was to offer valuable insights that can guide clinicians in determining the optimal age for surgical treatment.

## Methods

### Patients

A retrospective analysis was performed on a cohort of infants aged up to 1 year who underwent surgery at our institution between January 2015 and December 2019. The diagnosis of these infants was established through a combination of physical examinations and confirmed by employing two or three methods, including barium enema, anorectal manometry, and intestinal biopsy. Additionally, all of the children received saline reflux enemas as part of their routine care for a duration of 2–3 weeks before surgery. Patients who were diagnosed with Hirschsprung’s allied disease (HAD) through postoperative pathologic findings, those who underwent staged surgery, and those who were lost to follow-up were excluded from the study.

In our hospital, patients underwent one-stage laparoscopic assisted pull-through surgery using modified Swenson technology. Preoperative bowel preparation typically involved colonic irrigation with normal saline before the surgery. Full-thickness or seromuscular mapping biopsies were done during the surgery to identify the precise location of the histologic transition zone. A frozen section was done to confirm ganglion cells in the suspected ganglionic bowel. If found, at least 5 cm of colon was removed to ensure the transition zone was taken out. If no ganglion cells were found, biopsies were taken 2 cm proximal to the original site until satisfactory results were achieved [12]. Transanal tubes were left in place for 7 days post-surgery to aid in bowel movements and prevent complications. Anal dilatation started at 14 days and continued for 3–6 months. Patients were monitored through phone, internet, or clinic visits.

The initial follow-up evaluation in our study was conducted no earlier than 3 years after the surgical procedure. The study participants were categorized into two groups according to the age at which they received surgical intervention: Group A, comprising patients who underwent surgery at 3 months of age or younger, and Group B, comprising patients who underwent surgery between 3 and 12 months of age. Prior to initiating the study, the research protocol received approval from the Ethics Committee of the Children’s Hospital of Chongqing Medical University.

### Measures and definitions

The collected data included various variables, such as the patient’s sex, age, type of HSCR, birth weight, weight at the time of surgery, duration of postoperative hospitalization, pre- and postoperative comorbidity with Hirschsprung-associated enterocolitis (HAEC), and additional postoperative complications, including obstructive symptoms, anastomotic leakage, and enterocolitis.

The children who comprised the study were subsequently monitored in 2022, and the outcomes of the functional evaluation were documented and examined. During the follow-up period, the collected data included the presence of additional congenital abnormalities, the extent of affected bowel, the occurrence of HAEC, the incidence of surgical complications, and the implementation or omission of surgical interventions. To facilitate the diagnosis of HAEC both preoperatively and postoperatively, it is recommended to consult the guidelines established by Gosain et al. for outlining the diagnostic and therapeutic approaches for HAEC [13]. Surgical complications were operationally defined as untowards occurrences that occurred following the surgical procedure and were causally linked to the initial surgical intervention, such as complications related to anaesthesia.

The researchers established communication with the subjects through either face-to-face interactions or telephone conversations. The assessment of functional bowel outcome was conducted by employing Rintala’s faecal incontinence score [14]. PedsQLTM4.0, which is a 23-item measurement model, was utilized to evaluate the health-related quality of life (QoL) of children aged 2 to 18 years. Parent proxy-report versions were employed for children aged 2 years and above to assess the domains of physical, emotional, school, and social QoL [15, 16].

### Statistics

The data are presented as percentages (%), medians (P25, P75), and means (SEMs), with a significance level of 0.05. The normality of continuous variables was assessed, and the t test was employed for normally distributed data, whereas the rank sum test was used for nonnormally distributed data. Categorical variables were analysed by using either the chi-square test or Fisher’s exact test. Statistical analysis was conducted by using SPSS 25.0 software. In this study, the Rintala scores were assessed by using the chi-square test or Fisher’s exact test, whereas the comparison of quality of life scores between groups was conducted by using the rank sum test (Mann-Whitney U test) due to the nonnormal distribution of the data.

## Results

### Patient demographics

A cohort of 235 children met the specified inclusion criteria, 139 (121 males and 18 females) of whom were under 3 months of age, and 96 (75 males and 21 females) of whom were over 3 months of age. Table 1 displays the demographic characteristics of the patients who were included in the research study.

**Table 1:** Clinical characteristics of two groups

	Under 3 months	Over 3 months	*P*
	*n* = 139	*n* = 96	
Male(%)	121(87%)	75(78.1%)	*P* = 0.083
Female(%)	18(12.9%)	21(221.8%)	
Type of HSCR n (%)			*P* = 0.766
Short segment type	17(12.2%)	9(9.3%)	
Typical segment type	93(66.9%)	68(70.8%)	
Long segment type	29(20.8%)	19(19.7%)	
Weight at birth(kg)	3.34(2.2–4.3)	3.26 (2.1–4.5)	*P* = 0.092
Age at surgery(months)	1.40(1.39–1.59)	8.15(7.13–8.16)	*P* < 0.001
Weight at surgery (kg)	3.31 (3.0-3.9)	8.0 (7.0–9.0)	*P* < 0.001
Operating time(min)	110(106.1-127.2)	130(120.7-142.8)	*P* < 0.001
Extent of resection of intestine(cm)	25(27.8–31.9)	37.1(35-43.4)	*P* < 0.001
Length of post-operative stay(d)	9.0(7.0–37.0)	7.0(7.0–14.0)	*P* < 0.001

Note: Ages, length of post-operative stay, extent of resection of intestine and operating times are expressed as medians with range

Student’s t test for continuous variables

Mann–Whitney test for nominal categories

The two groups exhibited similarities in terms of sex (*P* = 0.083) and type of HSCR (*P* = 0.766). The mean birth weight was 3.34 kg in the group aged under 3 months and 3.26 kg in the group aged over 3 months (*P* = 0.092). The median age at surgery was 1.4 months in the under-3-month group and 8.15 months in the over-3-month group (*P* < 0.001). The under-3-month group had a relatively shorter surgery time than did the over-3-month group, with a median time of 110 min versus 130 min, respectively (*P* < 0.001). Furthermore, the under-3-month group exhibited a comparatively shorter resected intestine length than the over 3-month group, with a median length of 25 cm in contrast to 37.1 cm, respectively (*P* < 0.001). Conversely, the postoperative hospitalization duration was longer in the under-3-month age group, with a median duration of 9.0 days compared to 7.0 days in the group consisting of individuals older than 3 months (Table 1).

### Functional bowel outcomes

The results of the statistical analysis of anal function in the two groups are shown in Table 2.

**Table 2:** Functional bowel outcomes

	Under 3 months	Over 3 months	Total	X²	*P*
	*n* = 138	*n* = 96			
**Ability to hold back defecation**				0.922	0.631
Always	54(39.1%)	43(44.8%)	97(41.5%)		
Problems < 1/wk	69(50.0%)	42(43.8%)	111(47.4%)		
Weekly problems	15(10.9%)	11(11.5%)	26(11.1%)		
No voluntary control	0	0	0		
**Feels/reports the urge to defecate**					0.432
Always	64(46.4%)	46(47.9%)	110(47.0%)		
Most of the time	63(45.7%)	38(39.6%)	101(43.2%)		
Uncertain	11(8.0%)	11(11.5%)	22(9.4%)		
Absent	0	1(1.0%)	1(0.4%)		
**Frequency of defecation**				6.077	0.048
Every other day to twice a day	119(86.2%)	73(76.0%)	192(82.1%)		
More often	12(8.7%)	19(19.8%)	31(13.2%)		
Less often	7(5.1%)	4(4.2%)	11(4.7%)		
S**oiling**				2.684	0.443
Never	11(8.0%)	8(8.3%)	19(8.1%)		
Staining < 1/wk, no change of underwear	58(42.0%)	50(52.1%)	108(46.2%)		
Frequent staining	63(45.7%)	34(35.4%)	97(41.5%)		
Daily soiling	6(4.3%)	4(4.2%)	10(4.3%)		
**Acccidents**					0.571
Never	108(78.3%)	80(83.3%)	188(80.3%)		
Fewer than 1/wk	29(21.0%)	15(15.6%)	44(18.8%)		
Weekly, require protect aids	1(0.7%)	1(1.0%)	2(0.9%)		
Daily	0	0	0		
**Constipation**					0.817
No constipation	91(65.9%)	66(68.8%)	157(67.1%)		
Manageable with diet	32(23.2%)	22(22.9%)	54(23.1%)		
Management with laxatives	13(9.4%)	8(8.3%)	21(9.0%)		
Management with enemes	2(1.4%)	0	2(0.9%)		
**Social problems**				4.333	0.115
No social problems	85(61.6%)	67(69.8%)	152(65.0%)		
Sometimes	42(30.4%)	18(18.8%)	60(25.6%)		
Problems restricting social life	11(8.0%)	11(11.5%)	22(9.4%)		
Severe social/psychological problems	0	0	0		
**Assessment of quality of life**					0.968
Normal(≥17)	62(44.9%)	46(47.9%)	108(46.0%)		
Good(13–16)	61(44.2%)	40(41.7%)	101(43%)		
Fair(9–12)	14(10.1%)	9(9.4%)	23(9.8%)		
Poor(≤8分)	1(0.7%)	1(1.0%)	2(0.9%)		

Note: One child in the under 3 months group had a colostomy because of postoperative complications, which was not closed at follow-up time

Chi-square test for nominal categories, and when the expected counts were less than 5, Fisher’s exact test was used

According to Rintala’s score, the proportion of individuals with normal anal function was 44.9% (62/138) in the group aged less than 3 months and 47.9% (46/96) in the group aged more than 3 months, with no statistically significant correlation observed between the two groups (*P* = 0.968). Regarding the capacity to regulate bowel movements, the percentage of children exhibiting good control was greater in the group aged more than 3 months than in the group aged less than 3 months (44.8% vs. 39.1%, respectively; *P* = 0.631). Regarding the capacity to restrain defecation, the group of children aged more than 3 months exhibited a greater proportion of consistent stool perception than did the group aged less than 3 months (47.9% vs. 46.4%, respectively; *P* = 0.432). Additionally, in terms of soiling, a greater percentage of children in the over 3 months age group than in the under 3 months age group did not experience soiling after surgery (8.3% vs. 8.0%, respectively; *P* = 0.443). Conversely, the group under 3 months of age had a greater proportion of children who experienced soiling compared to the group over 3 months of age (50% vs. 39.6%, respectively; *P* = 0.443), as well as more frequent instances of soiling. The prevalence of incontinence was 78.3% among individuals aged under 3 months and 83.3% among those aged over 3 months. In general, the postoperative defecation function of both groups was deemed to be satisfactory. Furthermore, the group over 3 months of age exhibited a greater percentage of excellent anal function than did the treatment group under 3 months of age, although this difference did not reach statistical significance (*P* > 0.05).

### Quality of life outcomes

**Fig. 1:**
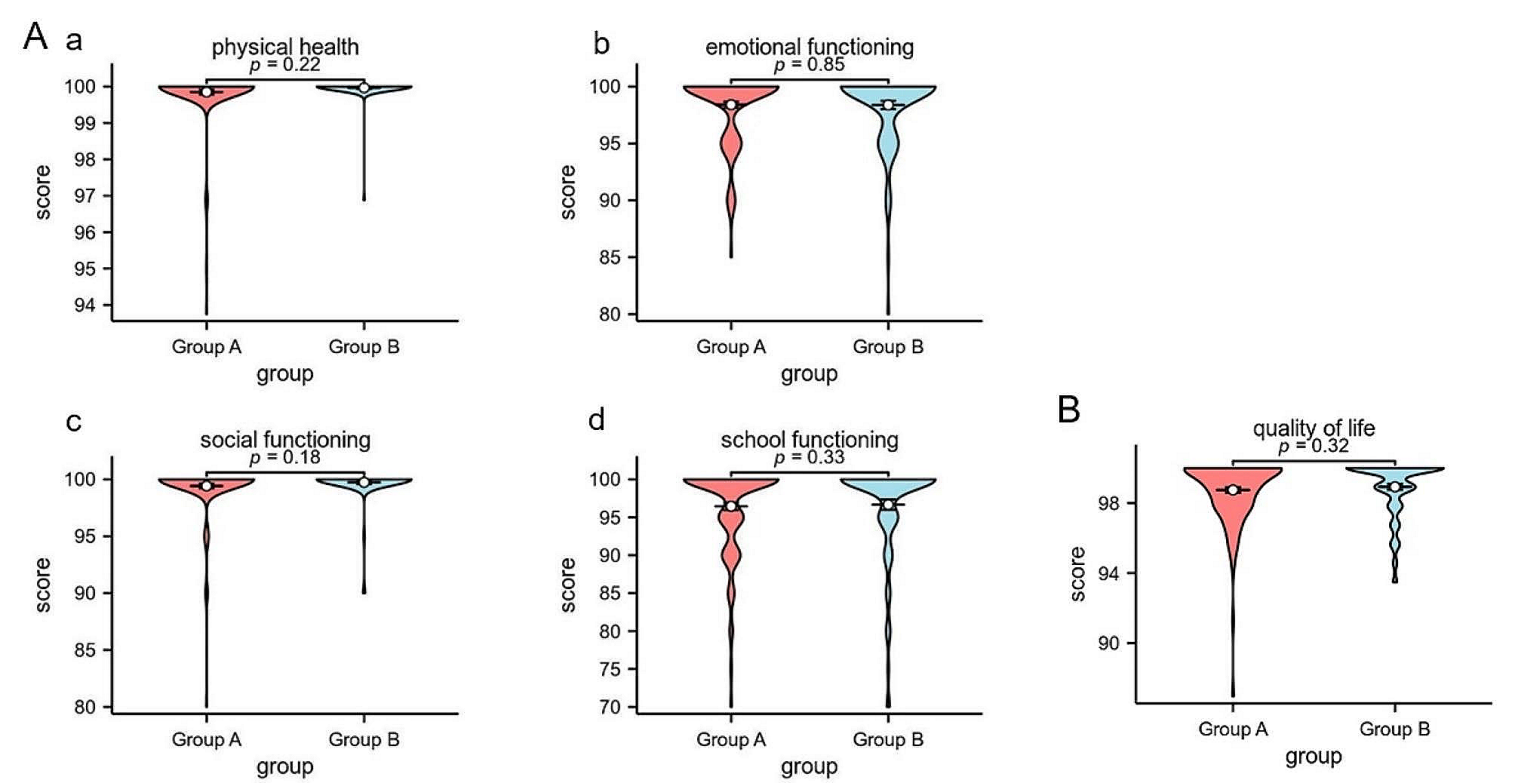
Quality of life assessment. **(A)** Categorization of Pediatric Quality of Life (PedsQL) 4.0. (a) physical health, (b)emotional functioning, (c)social functioning, and (d)school functioning. **(B)** Total score of PedsQLTM 4.0. Mann–Whitney test for nominal categories. Group A: Under 3 months of age group. Group B: Over 3 months of age group

This study involved a quality of life assessment questionnaire (the PedsQLTM 4.0 scale) for all of the children. The scale was utilized for children aged 2 years and above. The obtained scores were then synthesized, taking into account the physical health, emotional functioning, social functioning, and school functioning scores. The analysis demonstrated that the medians of these scores were comparable between the two groups of children. Additionally, the distribution range was similar, and no statistically significant disparities were observed between the groups in terms of physical health, emotional functioning, social functioning, or school functioning scores (Fig. 1A). In relation to quality of life, the distribution of scores exhibited a greater level of concentration and lacked a statistically significant positive correlation within the group aged over 3 months compared to the other group (*P* = 0.32) (Fig. 1B).

#### Postoperative complications

According to the data presented in Table 3, within one year following surgery, three infants aged under three months in the cohort sought medical interventions for intestinal obstruction. Among them, one child experienced relief through conservative treatment, whereas the remaining two children underwent temporary colostomy due to persistent abdominal distension that could not be alleviated. Subsequently, these two children exhibited normal defecation through an artificial anus, and the stoma was closed within six months postsurgery. Notably, all three infants achieved more satisfactory anal function during the follow-up examination. Notably, within a span of one month postsurgery, two children exhibited an occurrence of anastomotic fistula. Subsequently, one of these individuals necessitated a secondary surgical intervention due to the presence of severe peritonitis, whereas the other child did not undergo surgery because her abdominal symptoms did not reach a critical level. Upon subsequent evaluation, it is worth mentioning that both children demonstrated the ability to maintain regular and normal bowel movements, thereby indicating a satisfactory quality of life. A total of four children (all older than 3 months) underwent surgical intervention for postoperative intestinal obstruction. One child experienced persistent abdominal distension even two years after the operation. Consequently, the child underwent intestinal stoma surgery and partial resection of the colon. At the time of follow-up, the stoma had not yet closed, and the child maintained regular bowel movements through the artificial anus, thus leading to a reasonable quality of life. Conversely, the remaining children exhibited more satisfactory anal function during the follow-up period. Within a span of three months postsurgery, a single child exhibited perianal abscess accompanied by dermatitis, which was subsequently ameliorated after the performance of the incision and drainage surgery.

**Table 3:** Postoperative complications

	Perianal dermatitis	Anal stenosis	Anastomotic stricture	Intestinal obstruction
**Under 3 months**	0	1(0.72%)	2(1.4%)	3(2.2%)
**Over 3 months**	1(1.0%)	0	0	4(4.2%)
**P**				*P* = 0.029

Note: Chi-square test for nominal categories, and when the expected counts were less than 5, Fisher’s exact test was used

The noteworthy observations in Table 4 pertain to the occurrence of HAEC. Prior to surgery, a substantial positive association was observed between the two groups. Specifically, 69.1% of the children younger than 3 months of age experienced HAEC before the operation, whereas only 30.9% experienced HAEC after the operation, and the difference was statistically significant (*P* < 0.001). In contrast, 27.1% of the children above 3 months of age had preoperative HAEC, and 28.1% had postoperative HAEC (*P* = 0.867).

**Table 4:** Incidence of HAEC

	Under 3 months	Over 3 months	Total	*P*
	*n* = 139	*n* = 96	*n* = 235	
**HAEC n(%)**				
**Pre-operation**	96(69.1)	26(27.1)	122	*P* < 0.001
**Post-operation**	43(30.9)	27(28.1)	70	*P* = 0.666
	*P* < 0.001	*P* = 0.867		

Note: Chi-square test for nominal categories, and when the expected counts were less than 5, Fisher’s exact test was used

Student‘s t-test was used for pre- and post-operative comparisons in the same treatment group

## Discussion

Since the conclusion of the 20th century, a plethora of studies have comprehensively documented the safety and feasibility of one-stage definitive surgery for HSCR. Subsequent postoperative follow-up has consistently indicated that one-stage surgery is correlated with superior intestinal function compared to staging surgery [8, 17]. Moreover, the emergence of minimally invasive surgery has resulted in a reduction in the mean age at which surgical procedures are performed. Nevertheless, the implications of surgical timing for long-term functionality have not yet garnered widespread recognition. This study demonstrated that, in comparison to the treatment group consisting of individuals aged 3 months and above, children under the age of 3 months exhibited shorter operation durations and underwent less extensive resection of the intestinal tube (*P* < 0.001). We hypothesized that this difference could be attributed to a smaller degree of bowel dilation and reduced faecal accumulation in neonatal patients than in children over 3 months of age. Additionally, children over 3 months of age experience a longer waiting time after diagnosis, thus possibly resulting in poorer bowel cleansing efficacy. This correspondingly leads to hypertrophy of the dilated segment of the intestinal tube, an increased range of intestinal tube resection during surgery, and heightened bleeding. Consequently, these factors undoubtedly contribute to increased pain and psychological pressure experienced by both the children and their parents.

In the current study, it was observed that the treatment group comprising individuals under 3 months of age exhibited a greater proportion of individuals capable of perceiving the urge to defecate than did the group consisting of individuals over 3 months of age (92.1% vs. 87.5%, respectively; *P* = 0.432). Furthermore, based on clinical expertise, meticulous intraoperative dissection of the mucosa located above the dentate line is deemed to be crucial for the maintenance of rectal sensation following surgery. In the present study, the prevalence of bowel control issues, soiling, and constipation during postoperative follow-up was comparatively greater among children aged younger than 3 months than among those aged older than 3 months (refer to Table 1). Nevertheless, numerous scholars attribute this phenomenon to the younger age at the time of surgery and posit that such a younger age is associated with an increased likelihood of diminished postoperative functionality in children [6, 18, 19]. According to the findings of Huang et al. [20], undergoing surgery before the age of two months is associated with an increased likelihood of experiencing postoperative faecal incontinence and urinary incontinence. This effect can be attributed to the anatomical constraint of a narrow anal opening and the incomplete development of defecation and urination reflexes during the neonatal period. Consequently, performing surgical procedures during this critical stage may not only impair these bodily functions but also increase the potential for complications. Moreover, children in this developmental stage exhibit decreased resistance to infections as a result of their underdeveloped active immunity and dependence on passive immunization through maternal antibodies. Additionally, the prevalence of Hirschsprung-associated enterocolitis (HAEC) is significantly elevated in this population due to persistent colorectal obstruction leading to malnutrition [13, 21].

Faecal soiling, which is a prominent long-term issue following HSCR, should not be regarded merely as a complication akin to constipation. Beyond the challenges of incontinence, the frequent occurrence of soiling can profoundly diminish the overall quality of life for affected patients. Notably, certain surgeons have observed noteworthy amelioration of soiling problems as patients have aged [22]. In the current investigation, it was observed that the occurrence and regularity of faecal soiling were more prevalent among children in the treatment group aged less than 3 months than among those in the treatment group aged more than 3 months (*P* = 0.443). This phenomenon can be attributed to various factors, including impairment of the internal sphincter and pelvic nerve, diminished sensory perception in the anal skin, and rectal dysfunction, among others [23]. The risk of intraoperative injury is increased due to the indistinct anatomical boundaries of the internal anal sphincter, whereas the external anal sphincter plays a crucial role in preventing postoperative faecal soiling [24].

In addition to evaluating anal function, comprehensive physical and psychological assessments are essential of assessing the quality of life in pediatric patients with HSCR. When research solely concentrates on quality of life pertaining to bowel dysfunction while disregarding the physical and psychosocial dimensions, it often results in study limitations. This is because most children with concurrent bowel dysfunction in the postoperative phase exhibit good adaptation and tend to experience an improved overall quality of life as they age [25, 26]. In this study, it was found that most of the children were able to perform daily physical activities and school life and had normal interactions and psychological activities with their peers. These findings were consistent with the results obtained from other studies using the PedsQLTM 4.0 scale [27, 28]. However, it should be noted that the children in the treatment group above 3 months of age exhibited higher levels of satisfaction with their quality of life. Conversely, no statistically significant difference was observed between the two groups in terms of quality of life based on disease level. It is important to acknowledge the fact that the study did not encompass children with the total colonic aganglionosis (TCA) and had relatively limited data on children with the long segment type. Therefore, it would be premature to conclude that age at surgery for HSCR does not impact postoperative quality of life. Hartman et al. [29]. observed a consistent trend in which parents assigned higher scores during the follow-up of both the child and themselves, which aligns with the findings of the current study. Consequently, for postoperative follow-up of the child, a comprehensive and desirable approach would involve incorporating both the parents’ assessment and the physician’s evaluation of the child’s symptoms.

Recent data suggest that the incidence of preoperative HAEC ranges from 6 to 60%, and that of postoperative HAEC ranges from 25–37% [13]. In the current study, the incidence of preoperative HAEC in children in the treatment group over 3 months of age was 27.1%, and the incidence of postoperative HAEC was 28.1%, which is generally in agreement with the results reported in the literature. The incidence of preoperative HAEC in children in the treatment group under 3 months of age was 69.1%, and the incidence of postoperative HAEC was 30.9%, which is inconsistent with the results of previous studies. This effect may be attributed to the following reasons. (a) There is inconsistency in the number of participants included in various studies. (b) The incidence of postoperative HAEC has decreased, which is possibly due to the routine practice of regular postoperative anal dilation in children at our institution. This preventative measure helps to prevent anastomotic strictures and defecation problems to some extent, thereby reducing the occurrence of HAEC [30, 31].

The increased incidence of HAEC prior to surgery, especially in the cohort of infants younger than 3 months, may be linked to immature mucosal immunity in neonates. This factor has been recognized as being a significant protective factor against the development of HAEC in comparison to older children [32]. This finding aligns with the earlier hypothesis suggesting that the combination of immature immunity resulting from delayed intestinal development and younger age contributes to the development of HAEC. Additionally, the suboptimal nutritional status and incomplete obstruction observed in younger children have been found to hinder the immune system, thereby impeding the ability to mount appropriate immune responses to alterations in the composition of the intestinal flora [33]. An alternative explanation is that the preoperative diagnosis of HAEC is predominantly based on data extracted from the child’s medical history, thus potentially introducing subjective biases. Some children who are referred to our institution experience a protracted diagnostic process as a result of technical and knowledge constraints at the initial hospital, thus resulting in a heightened incidence of HAEC due to delays in receiving appropriate and specific treatment. In addition, the incidence of postoperative HAEC was greater in the Group A than in the Group B (30.9% vs. 28.1%), which may be due to the smaller diameter of the neonatal intestinal canal, and small-diameter anastomosis is prone to localized stenosis, which is also an important factor for the high incidence of HAEC. The exact mechanism by which HAEC occurs is currently unknown. Several studies have identified a correlation between gastrointestinal mucosal immunity and dysbiosis of particular microbiota in the gut, thereby indicating the existence of specific bacterial colonies that may contribute to the susceptibility of children to recurrent HAEC [34, 35]. According to the findings of the present study, our institution recommends early surgical intervention for children diagnosed with conditions that do not involve serious complications or severe malnutrition, with generally favourable postoperative outcomes. One of the primary benefits of surgical intervention during the neonatal period is the prevention of preoperative complications, particularly in paediatric patients with Hirschsprung’s disease. This intervention helps to prevent the prolonged retention of faecal matter in the intestines due to inadequate defecation, thereby reducing the likelihood of developing Hirschsprung-associated enterocolitis (HAEC) to some degree [17, 36]. However, this conclusion does not indicate that surgical treatment can completely eliminate HAEC. It has been previously documented that it can still occur in the remaining bowel of children with HSCR due to immunological abnormalities [37, 38].

This study had several limitations. In our clinical practice, it is customary to administer a minimum of 2 weeks of reflux enema treatment followed by early surgical intervention for children who have been diagnosed with the nontotal colonic type based on rectal mucosal biopsy. However, the findings of this study do not indicate a significant correlation between early surgery and improved mid-term prognosis. This lack of association may be attributed to several factors. First, the limited number of patients who were included in this retrospective study, which was conducted at a single centre, may have introduced bias into the statistical analysis. Therefore, it is imperative to conduct large-scale, multicentre prospective studies to further elucidate these findings. Second, it should be noted that the utilization of the PedsQLTM 4.0 Quality of Life Rating Scale in this particular study was limited to children aged 2 years and older. This restriction resulted in disruptions during the postoperative follow-up of the children and hindered the ability to conduct an unbiased and all-encompassing evaluation of their postoperative recovery. Additionally, it is imperative to acknowledge that the number of outpatient visits for postoperative follow-up varied among the children due to the diverse progression of their respective diseases. These aspects should be taken into consideration when interpreting the findings of this study. Finally, the current clinical demarcations for the graded diagnosis of HAEC lack clarity, and the reliance on parental judgement in the assessment of bowel function and quality of life in this study introduces potential bias in the results. Consequently, it is imperative to integrate more dependable and comprehensive diagnostic scoring mechanisms into the diagnostic criteria for HAEC.

## Conclusion

This current study represents the first attempt to examine the influence of age at surgery on the mid-term clinical outcomes of one stage laparoscopic assisted surgery for HSCR during infancy, thus utilizing a substantial sample size. In this study, the bowel function and quality of life of children aged 3–7 years were assessed and correlated with the age at surgery. This study did not identify any discernible variation in the effects of age at surgery on postoperative anal function and quality of life in children. However, within the treatment group comprising children aged under 3 months in this study, a notable disparity in the occurrence of HACE was observed before and after surgery, thus suggesting that early surgical intervention may prove efficacious in averting the progression of HAEC and mitigating the disease’s exacerbation in infants. To summarize, prompt surgical intervention for definitively diagnosed HSCR during infancy offers the benefits of reduced operative duration, diminished intraoperative harm, decreased incidence of HAEC, and generally satisfactory postoperative anal function and quality of life.

## Data Availability

Data can be made available on request to corresponding author.

## Abbreviations

HSCR: Hirschsprung’s disease
HAEC: Hirschsprung-associated enterocolitis
TEPT: Transanal endorectal pull-through
HAD: Hirschsprung’s allied disease
QoL: Quality of life
